# Geochemical Control of PAHs by Inflowing River Water to West Nanao Bay, Japan, and Its Influences on Ecological Risk: Small-Scale Changes Observed under Near-Background Conditions at an Enclosed Bay

**DOI:** 10.3390/ijerph181910310

**Published:** 2021-09-30

**Authors:** Rodrigo Mundo, Tetsuya Matsunaka, Hisanori Iwai, Shinya Ochiai, Seiya Nagao

**Affiliations:** 1 Division of Material Chemistry, Graduate School of Natural Science and Technology, Kanazawa University, Kanazawa 920-1192, Japan; rodrigomundo12@gmail.com; 2 Low Level Radioactivity Laboratory, Institute of Nature and Environmental Technology, Kanazawa University, Nomi 923-1224, Japan; h-iwai@se.kanazawa-u.ac.jp (H.I.); sochiai@se.kanazawa-u.ac.jp (S.O.); seiya-nagao@se.kanazawa-u.ac.jp (S.N.)

**Keywords:** polycyclic aromatic hydrocarbons, remote coastal marine areas, environmental organic pollutants, ecological risk assessment

## Abstract

Polycyclic aromatic hydrocarbons (PAHs), even at low concentrations, have been shown to trigger changes in life cycles and provoke abnormal behaviors in numerous marine organisms. From May 2019 to September 2020, particulate and dissolved PAH concentrations were analyzed on the surface water of West Nanao Bay, Japan, to determinate their levels, emission sources, environmental pathways, and ecological risks at this remote but semi-enclosed bay. The 14 targeted PAHs were analyzed by HPLC-fluorescence detector. Mean total PAH concentrations were lower than 20.0 ng L^−1^ for most samples. Based on fluoranthene (Flu) to pyrene (Pyr) ([Flu]/[Flu + Pyr]) and benzo[a]anthracene (BaA) to chrysene (Chr) ([BaA]/[BaA + Chr]) isomeric ratios and a varimax rotated PCA, it was established that biomass combustion was the principal source in the particulate phase and that liquid fossil fuel combustion was the principal source in the dissolved phase. From salinity and turbidity distribution, riverine discharges were determined to be the major and continuous transportation pathway of particulate PAHs. It was observed that rain events had a role in the transport of dissolved PAHs. The risk quotients (RQ_∑14_ _PAHs_ _(NCs)_: 0–84.53) indicated that PAHs represented a very low to low acute environmental risk. The results of this study will contribute to filling the paradigm gap of ecotoxicological studies in remote areas, working as a booster for future in-lab studies of non-lethal implications of endocrine disruptors such as PAHs.

## 1. Introduction

Polycyclic aromatic hydrocarbons (PAHs), released to the environment mainly from the incomplete combustion of fossil fuels and biomass as well as from the leakage of petroleum derivates [1,2], are hazardous pollutants with adverse carcinogenic and/or mutagenic potential [3]. PAHs, even at low concentrations, have been shown to trigger changes in life cycles and to provoke abnormal behaviors in numerous marine organisms [4,5,6,7,8]. As mainly a hormone-regulated process, fish reproduction is very sensitive to physical and chemical stressors, such as PAHs [9]. Nicolas (1999) summarized the direct effects of PAHs in increasing vitellogenesis in various species of fish [4]. Recent studies have continued developing in this area [10,11,12,13,14,15]. Vitellogenesis in most species of fish occurs only once in the reproductive cycle. Consequently, even in non-lethal concentrations, PAHs have the potential to affect the whole reproductive cycle of the organism, leading to a gradual decrease in the population [4,11,12,13,14]. Additionally, PAHs can provoke “behavioral toxicity” syndromes categorized by change in activeness and responsiveness [16]. The exposure to PAHs (total concentration: 5–50 μg L^−1^) was reported to caused statistically significant reductions in the swimming activity of larval killifish during the high activity dark periods (*p* < 0.05) [17]. The toxicity in invertebrate marine animals has also been widely studied [18,19,20,21]. For instance, the exposure of oyster (Crassostrea gigas) embryos to benzo(a)pyrene at concentrations of 50 ng L^−1^ would increase abnormality rates from 30% to 45% [18].

Remote areas historically have been excluded from ecotoxicology paradigms, and thus there has been a lack of field data for realistic in-lab studies. For a deeper understanding regarding the chronic effects of PAHs in difficult-to-reach environments with low contamination levels (e.g., wildlife protected coastal areas), field measurements of PAHs in such environments are indispensable. Efforts are being made around the world to measure the extent of contamination, and reliable data are crucial for setting realistic goals in the fight to reduce anthropogenic stressors [22,23]. In addition, to improving remediation initiatives, it is necessary to understand the pollutants’ environmental pathways—that is, to understand the spatial distribution of pollutants and their propagation, considering different seasonal variables.

West Nanao Bay, in the Noto Peninsula, was selected not only for being a semi-enclosed and shallow water body, with longer water retention times and less influence from the Tsushima Warm Current than other coastal areas of the Sea of Japan [24], but also for being an important oyster production area, with a prefectural annual production of over 1400 tons [25], generating 400 direct jobs just for production [26]. West Nanao Bay is also home to many benthic organisms, and it is an important spawning ground for many fish species [27]. As a semi-enclosed bay, West Nanao Bay is expected to be a sink for anthropogenic pollutants, increasing the potential risk compared with other remote areas.

By studying PAH dynamics in West Nanao Bay, valuable data on a Japanese background coastal site were obtained. Reliable data will support legislation on anthropogenic stressors in wildlife protected coastal areas, such as RAMSAR sites, all around the world.

Specifically, this study aimed to elucidate (1) levels, (2) emission sources, (3) environmental pathways, and (4) ecological risks of PAHs in West Nanao Bay, Noto Peninsula, through the seasonal monitoring of the spatial distribution, as well as phase partitioning, of PAHs in surface seawater. Seasonal sampling was conducted between May 2019 and September 2020.

## 2. Materials and Methods

### 2.1. Study Area and Water Sampling

**Figure 1:**
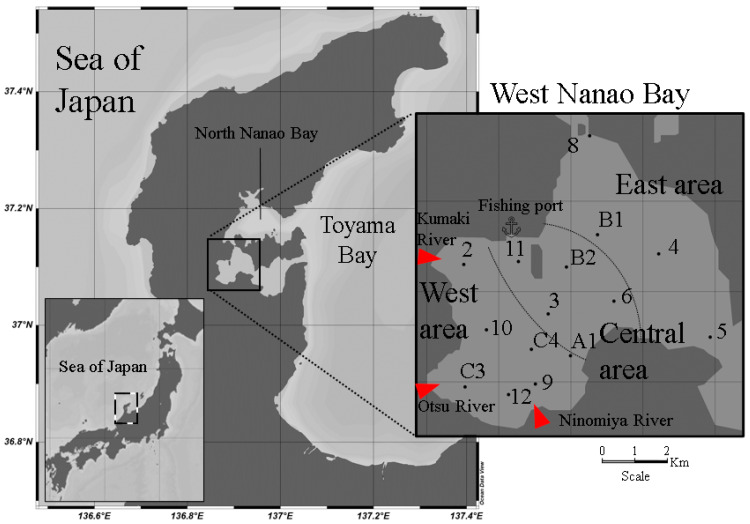
Sampling points at West Nanao Bay, Noto Peninsula, Japan. May 2019–September 2020.

West Nanao Bay is the innermost segment of Nanao Bay, isolated from Toyama Bay’s waters by Noto Island. For this study, West Nanao Bay was divided into three subareas: west, central, and east areas (Figure 1). The Kumaki, Otsu, and Ninomiya rivers flow into the west area of the bay, generating an estuary-like environment with shallow water (2–3 m). Fresh water discharges result in salinity that oscillates between 30 and 32 PSU. The seabed in this area is characterized by fine-grained (mud) sediments rich in organic matter and abundant in seagrass (*Zostera marina*) during the warm months. In the east area of the bay, the lack of seagrass, a sandier seafloor, and deeper waters (>10 m) are characteristic [24]. Salinity in the east area is usually over 32 PSU. Geologically, the central area presents the characteristic of being a transition of the previously described west and east subareas.

A set of 10 L surface seawater samples were collected from 15 points in West Nanao Bay (Figure 1) every three months from May 2019 to September 2020. Additional samples (10 L) were taken in August 2020 and June 2021 at Kumaki, Otsu, and Ninomiya River mouths (salinity 0–15 PSU) and at the nearest fishing port to sampling point 11 (37.114475 °N, 136.883191 °E). Samples were collected and stored in polished stainless well-closed containers. In addition to the sample from the fishing port, no oil slicks were visible on the water surface during the surveys. Water quality, such as water temperature, salinity, and turbidity, were measured on-site with a direct-reading comprehensive water quality meter (AAQ171, JFE Advantech, Hyogo, Japan) at a depth of 0.5 m. Daily precipitation data were obtained from the nearest monitoring station (Nishiyachi), located upstream of the Kumaki River and operated by the Ishikawa Prefectural Civil Engineering Department River Division.

### 2.2. Reagents and Chemicals

Extraction recoveries were quantitatively determined by an internal standard mix (ISTD) confirmed by two deuterated PAHs in phenanthrene (Phe-*d*_10_) and pyrene (Pyr-*d*_10_). Supelco EPA 610 PAH Mix was used as the reference standard for the target PAHs. All reagents were at least analytical reagent grade. Specific suppliers and purity grades were previously reported in Mundo et al. (2020) [28].

### 2.3. Pretreatment and Measurements of PAHs

Supelco EPA 610 PAH Mix was utilized as reference to determine 14 out of the 16 US EPA-priority PAHs. Concentrations of acenaphthene (Ace), fluorene (Fle), anthracene (Ant), phenanthrene (Phe), fluoranthene (Flu), pyrene (Pyr), benzo[a]anthracene (BaA), chrysene (Chr), benzo[b]fluoranthene (BbF), benzo[k]fluoranthene (BkF), benzo[a]pyrene (BaP), benzo[ghi]perylene (BPe), indeno[1,2,3-cd]pyrene (IDP), and dibenzo[a,h]anthracene (DBA) were analyzed. The samples’ pretreatment procedures were conducted as described in previous studies [28,29,30]. Briefly, particulate phase PAHs and dissolved phase PAHs were simultaneously separated by tandem filtration through 0.5 µm glass-fiber filters (GC50, Advantec, Hyogo, Japan) and C18 (octadecyl) disks (Empore, St. Paul, MN, USA) at a flow rate of <200 mL min^−1^ (Figure S1). After being air-dried, the glass fiber filters were dried over silica gel in the dark. Filters were stored at −20 °C until extraction with 100 mL dichloromethane. Dimethyl sulfoxide (DMSO; 100 μL) was added before concentration. Samples were finally reconstituted to 1 mL with acetonitrile. After the resulting solution was filtered with a membrane disk (HLC-DISK3, pore size 0.45 μm, Kanto Chemical Co., Tokyo, Japan), PAHs were separated and characterized by HPLC-fluorescence analysis. HPLC-fluorescence analysis of PAHs is described in detail in the Supplementary Material.

### 2.4. Quality Control

Method recoveries, including measurement sensitivities for HPLC assay of each sample, were estimated using internal standards of deuterated analogues of phenanthrene (Phe-*d*_10_) and pyrene (Pyr-*d*_10_), with which samples were spiked at the ultrasonic extraction stage for particulate PAHs and after seawater samples were filtered by C18 disks for dissolved PAHs. Overall recovery rates for dissolved PAHs’ internal standards were 80.3 ± 13.3% for Phe-*d*_10_ and 88.6 ± 14.2% for Pyr-*d*_10_. Recovery rates for particulate PAHs’ internal standards were 95.3 ± 10.1% for Phe-*d*_10_ and 96.6 ± 16.2% for Pyr-*d*_10_. To calculate the loss due to extraction procedures, recoveries of Phe-*d*_10_ and Pry-*d*_10_ were utilized for 3 rings- and 4–6 rings PAHs, respectively.

Background derived from pretreatment and measurements of PAHs were measured several times during the current research, and an average of all measurements were subtracted from sample concentrations. For each background measurement, blank tests were performed with 100 mL of ultra-pure water. The background concentrations in 10 L samples were Σ_14_PAH_part_ = 0.21 ng L^−1^ and Σ_14_PAH_diss_ = 0.27 ng L^−1^.

### 2.5. PAH Source Estimation

PAH compositions vary among petrogenic, pyrogenic, biogenic, and diagenetic sources, although all of them can be found naturally in many areas of the world after specific events such as petroleum seeps and forest fires. Nowadays, petrogenic and pyrogenic events are mainly anthropogenic, releasing great amounts of PAHs to the environment and expanding their distribution ranges even to remote areas such as the Arctic and the Antarctic [31,32,33]. Their emissions origins, such as petrogenic and pyrogenic, are commonly estimated by using molecular indices based on ratios of PAH concentrations, according to the relative thermodynamic stabilities of each pair [34,35,36].

A [Flu]/[Flu + Pyr] ratio less than 0.4 indicates the predominance of petrogenic sources, while a range between 0.4 and 0.5 suggests the dominance of fuel combustion processes, and values greater than this suggest grass, coal, and wood combustion. [BaA]/[BaA + Chr] ratios lower than 0.2 suggest petrogenic origins, values between 0.2 and 0.35 suggest automobile emissions, and values greater than 0.35 would suggest biomass combustion [37].

Principal component analysis (PCA) was additionally performed to clarify the discrepancies that arose from the diagnostic ratios method [38,39,40,41]. A varimax rotation and normalization was performed so the obtained data matrix would be comparable with those in previous studies. The rotation is commonly used to maximize the sum of the variances of the squared loadings within principal components and so to simplify the expression in terms of fewer PAHs each. The new data set obtained after varimax rotation contained fewer non-zero weights, which made the results easier to interpret and compare among studies.

### 2.6. Ecological Risk Assessment

The potential ecological risk of a given PAH was estimated by its risk quotients (RQ) for aquatic organisms [42,43,44,45]:

(1) $${\mathrm{RQ}}_{i}={\left[\mathrm{PAH}\right]}_{i}/{\mathrm{QV}}_{i}$$

where [PAH]_i_ and QV_i_ are the total concentration of the detected PAH in each water sample and the corresponding quality value, respectively. Two sets of quality values—the negligible concentrations (NCs) and the maximum permissible concentrations (MPCs)—were calculated by Kalf et al. [43]. Therefore, for any given PAH, the values of RQ_NCs(i)_ and RQ_MPCs(i)_ can be defined as follows:

(2) $${\mathrm{RQ}}_{\mathrm{NCs}\left(i\right)}={\left[\mathrm{PAH}\right]}_{i}/{\mathrm{QV}}_{\mathrm{NCs}\left(i\right)}$$

(3) $${\mathrm{RQ}}_{\mathrm{MPCs}\left(i\right)}={\left[\mathrm{PAH}\right]}_{i}/{\mathrm{QV}}_{\mathrm{MPCs}\left(i\right)}$$

where RQ_NCs(i)_ and RQ_MPCs(i)_ represent the quality value of the NCs and MPCs for the given PAH, respectively.

Based on RQ_NCs_ and RQ_MPCs_ values, risk assignment parameters were determined [44]. Quality values for ten of the sixteen USEPA priority PAHs were originally reported [43]. For the remaining six, toxic equivalent factors (TEFs) were utilized to define the missing quality factors [46]. PAHs with similar TEFs should have similar negligible concentrations (NCs) and maximum permissible concentrations (MPCs).

To determine the overall risk of the 14 studied PAHs, the RQ values for ∑_14_PAHs was calculated as defined by Cao et al. (2010) [45]:

(4) $${\mathrm{RQ}}_{\sum \mathrm{PAHs}\left(\mathrm{NCs}\right)}=\overset{14}{\underset{i=1}{\sum }}{\mathrm{RQ}}_{i\left(\mathrm{NCs}\right)}\;\left({\mathrm{RQ}}_{i\left(\mathrm{NCs}\right)}\geq 1\right)\;$$

(5) $${\mathrm{RQ}}_{\sum \mathrm{PAHs}\left(\mathrm{MPCs}\right)}=\overset{14}{\underset{i=1}{\sum }}{\mathrm{RQ}}_{i\left(\mathrm{MPCs}\right)}\;\left({\mathrm{RQ}}_{i\left(\mathrm{MPCs}\right)}\geq 1\right)\;$$

## 3. Results and Discussion

### 3.1. Oceanographical Conditions

**Figure 2:**
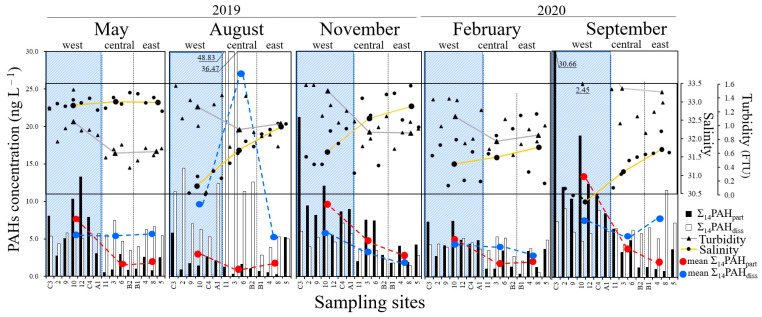
Variations in particulate (Σ_14_PAH_part_, black) and dissolved (Σ_14_PAH_diss_, white) PAHs in West Nanao Bay, Japan; May 2019–September 2020 and respective effluent rivers. Mean Σ_14_PAH_part_ and Σ_14_PAH_diss_ by area is marked in red and blue, mean turbidity (FTU) and salinity by area is marked in gray and yellow, respectively. Turbidity and salinity of each sampling point are marked by white triangles and white circles, respectively.

Salinity and turbidity average values at each subarea are summarized for each sampling season in Table 1. In Figures S2 and S3, the surface distribution of salinity and turbidity of the five sampling seasons are respectively presented. The west area, representing the riverine discharges, presented the lowest salinity in all five sampling seasons (Table 1, Figure 2). Turbidity presented an exact opposite trend, decreasing when moving away from the west area (Table 1, Figure 2). Tsushima warm current salinity varied between 31.6 and 35 PSU throughout the year, with salinity below 32 PSU in summer to autumn and above 34 PSU in winter to spring [47]. In West Nanao Bay, salinity was certainly lowest in summer (30.5–32.5), but it was never above 33.5 PSU (<32.5 in August, February, and September and <33.5 in May, November). As observed in Table 1, salinity in each subarea of the bay was lowest in summer and winter due to rainfall and snowfall, supported by the summer and winter monsoons [30]. The salinity trends observed in the present study support the observations of Yamazi, who found that oceanographical conditions in West Nanao Bay were largely controlled by riverine outflows and North Nanao Bay’s seawater and were not directly affected by the Tsushima Warm Current [24].

### 3.2. PAH Levels

**Figure 3:**
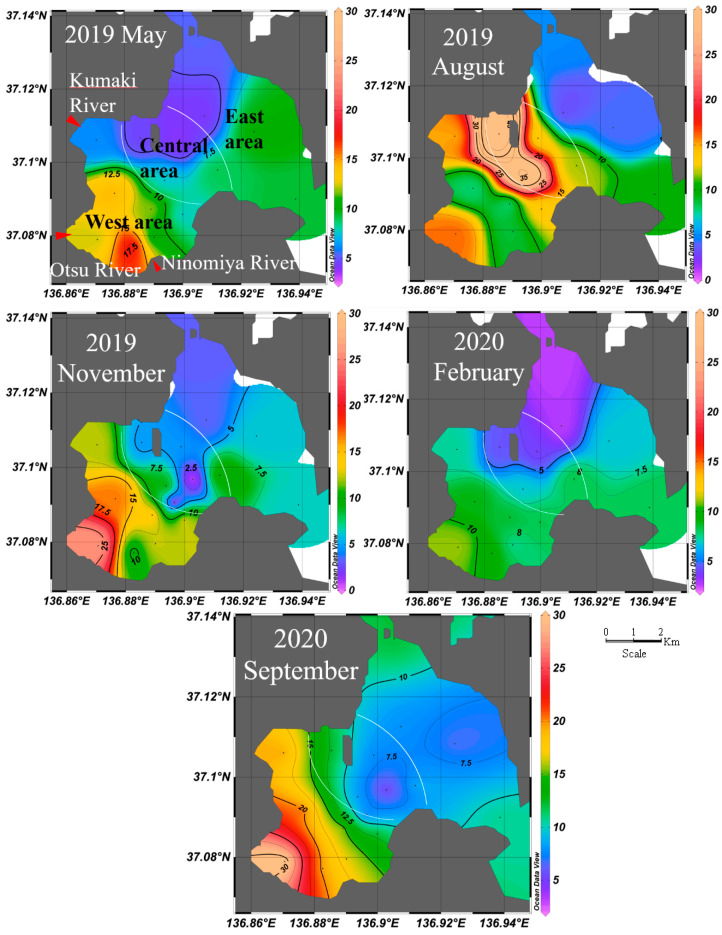
Spatial distribution of total PAH (Σ14PAHs) (particulate + dissolved) concentrations in surface water of West Nanao Bay, Japan; May 2019–September 2020.

Figure 2 and Figure 3 show the variations in Σ_14_PAH_part_ and Σ_14_PAH_diss_ in West Nanao Bay. The detailed values of Σ_14_PAH_part_ and Σ_14_PAH_diss_ for each sample are summarized in Table S1. The Σ_14_PAHs varied from 1.93 to 49.94 ng L^−1^ (0.19–30.66 ng L^−1^ for Σ_14_PAH_part_, 0.60–48.83 ng L^−1^ for Σ_14_PAH_diss_), with the maximum value measured at point 11 in August 2019. The mean Σ_14_PAHs (Σ_14_PAH_part_, Σ_14_PAH_diss_) were correspondingly 9.04 ng L^−1^ (4.03 ng L^−1^, 5.01 ng L^−1^) in May 2019, 14.37 ng L^−1^ (1.69 ng L^−1^, 12.69 ng L^−1^) in August 2019, 10.01 ng L^−1^ (6.66 ng L^−1^, 3.35 ng L^−1^) in November 2019, 7.35 ng L^−1^ (3.46 ng L^−1^, 3.89 ng L^−1^) in February 2020, and 15.22 ng L^−1^ (8.45 ng L^−1^, 6.77 ng L^−1^) in September 2020. The observed levels of PAHs at West Nanao Bay are comparable with other remote areas in Singapore, the northern part of Japan, and the Russian Far East, summarized elsewhere [28]. The 3- and 4-ring PAHs were the dominant species in dissolved PAHs, of which the sum of Fle, Phe, Flu, and Pyr occupied more than 80% of Σ_14_PAH_diss_ in all samples. For particulate PAHs, 4-, 5- and 6-ring PAHs were the major components, yet a more homogeneous distribution was observed in which each one of Flu, Pyr, BaA, Chr, BbF, BkF, BaP, DBA, BPe, and IDP occupied ±10% of Σ_14_PAH_part_ in all samples. The 5- and 6-ring PAHs were distributed mainly in the particulate phase, with almost negligible amounts in the dissolved phase. The difference in composition between particulate and dissolved PAHs is explained by the greater water solubility of PAHs with decreasing molecular size, due to the differences in water–octanol partition coefficients [29,34,48,49]. Examined by geographical subareas, the highest Σ_14_PAHs in the west area, decreasing towards the lowest Σ_14_PAHs in the east area, was also the general trend in August 2019, when abnormally high Σ_14_PAHs levels were detected in the central area of the bay (Figure 2).

The all-points average Σ_14_PAHs was 11.01 ng L^−1^, of which 43.9% was composed by the particulate phase, and 56.1% was the dissolved phase. In the west area, the all-points mean Σ_14_PAHs was 13.52 ng L^−1^, of which the particulate phase occupied 58.1%, and the dissolved phase occupied 41.9%. In the central area, the annual mean Σ_14_PAHs was 11.42 ng L^−1^, with 22.0% particulate phase and 78.0% dissolved phase. In the east area, the annual mean Σ_14_PAHs was 6.13 ng L^−1^, with 30.2% particulate phase and 69.8% dissolved phase. Particulate PAHs were dominant in the west area, while in the central and east area, dissolved PAHs dominated. Mean Σ_14_PAH_part_ was also highest in the west area, decreasing towards the central and east areas, although the mean Σ_14_PAH_diss_ did not always follow such a pattern (Table 1, Figure 2). Analyzed by sampling survey, the particulate phase percentage of Σ_14_PAHs varied between 44% and 64% in all surveys except for August, when particulate PAHs occupied no more than 9.3%.

Σ_14_PAHs (Σ_14_PAH_part_, Σ_14_PAH_diss_) for end members of West Nanao Bay are summarized in Table S2. Σ_14_PAHs (Σ_14_PAH_part_, Σ_14_PAH_diss_) for Kumaki, Otsu, and Ninomiya river during normal flow conditions (non-rain) were 18.13 ng L^−1^ (1.12 ng L^−1^, 17.71 ng L^−1^), 15.01 ng L^−1^ (9.52 ng L^−1^, 12.27 ng L^−1^), and 22.16 ng L^−1^ (0.46 ng L^−1^, 20.56 ng L^−1^), respectively. Σ_14_PAHs (Σ_14_PAH_part_, Σ_14_PAH_diss_) for the same river mouths during high flow conditions (during rain) were 136.78 ng L^−1^ (85.02 ng L^−1^, 51.76 ng L^−1^), 52.64 ng L^−1^ (11.85 ng L^−1^, 40.79 ng L^−1^), and 50.78 ng L^−1^ (6.04 ng L^−1^, 44.74 ng L^−1^), respectively. Σ_14_PAHs (Σ_14_PAH_part_, Σ_14_PAH_diss_) at the fishing port sample was 24.80 ng L^−1^ (4.41 ng L^−1^, 20.39 ng L^−1^).

### 3.3. PAH Emission Sources Characterization

#### 3.3.1. Diagnostic Ratios

**Figure 4:**
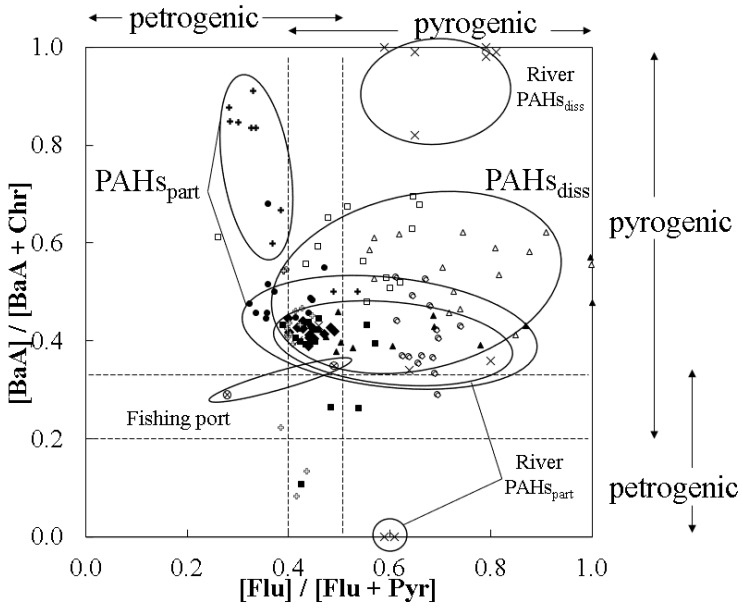
Cross plots of PAH diagnostic ratios: benzo[a]anthracene (BaA) to chrysene (Chr) ([BaA]/[BaA + Chr]) versus fluoranthene (Flu) to pyrene ([Flu]/[Flu + Pyr]). Open and filled symbols represent the values of particulate and dissolved PAHs, respectively. Triangles, squares, circles, diamonds, and crosses represent the data in May 2019, August 2019, November 2019, February 2020, and September 2020, respectively.

By using molecular indices based on ratios of PAH concentrations, emissions origins can be differentiated by petrogenic or pyrogenic origins; the results of the [BaA]/[BaA + Chr] versus [Flu]/[Flu + Pyr] plot is presented in Figure 4. Diagnostic ratio averages are presented separately by sampling season (Table 2) but not by sampling subarea because no considerable differences were observed.

The [Flu]/[Flu + Pyr] ratio during the sampled period ranged from 0.35 to 0.75 for most samples in the particulate phase and from 0.40 to 0.85 for most samples in the dissolved one. The particulate phase (mean: 0.49) was more influenced by liquid fossil fuel combustion, and the dissolved phase (mean: 0.62) was more influenced by biomass or coal combustion. The [BaA]/[BaA + Chr] in the particulate phase (0.10–0.50) had a mean of 0.43, which indicates a mixture between combustion and low temperature diagenesis, and a mean of 0.54 in the dissolved phase (0.30 to 0.90 for most samples), which would suggest biomass or coal combustion. Most river samples had a pyrogenic character, with ([BaA]/[BaA + Chr] and [Flu]/[Flu + Pyr]) ratio pairs of (0.34–0.43, 0.42–0.80) and (0.82–1.00, 0.59–0.81) for particulate and dissolved phases, respectively. In two particulate phase samples of Kumaki and Ninomiya rivers, under rainless conditions, the BaA concentration was below the detection limit. During fishing port sampling, oil slicks were visible on the water surface, and the dissolved phase effectively had a petrogenic character ([BaA]/[BaA + Chr] = 0.29, [Flu]/[Flu + Pyr] = 0.28). The particulate phase in the fishing port sample had a pyrogenic character ([BaA]/[BaA + Chr] = 0.35, [Flu]/[Flu + Pyr] = 0.49). It must be noted that some isomer pairs, such as in BaA–Chr, with considerable differences in photo-degradation rates can affect the reliability of the original source appointment method. Additionally, since the method is based on statistical analysis, the PAH sources directly interfering with the studied area may not be properly reflected within the set ranges.

#### 3.3.2. Principal Component Analysis

PCA is one of the most common statistical methods for source apportionment of PAHs. PCA was separately performed for particulate and dissolved phases, with 59 samples and 14 PAHs each. The component’s loading matrix is shown in Table 3. For each dissolved and particulate sample, two principal components were selected, so that 91.7% and 80.1% of the total variability would be explained in the dissolved and particulate phase, respectively.

The first component (PC1) in the particulate phase is highly loaded by 4–6 ring PAHs (Pyr, Flu, BbF, BPe, BaA, Chr, BkF, Ant, and IDP). PC1 is responsible for 71.0% of the total variance. This distribution is characteristic of pyrogenic events fueled by biomass [39]. The second component (PC2) of particulate phase is responsible for 9.2% of total variance and is loaded by 3-ring PAHs (Ace, Fle) and one 5-ring PAH (DBA). The loading by low molecular weight PAHs is an indication of petrogenic sources. The PC1 for the dissolved phase is responsible for 84.5% of the total variance. The marked presence of 3-ring PAHs (Ace, Fle, Phe, Ant) would indicate a certain petrogenic character, but the presence of higher molecular weight PAHs is characteristic for fossil fuel-powered pyrogenic events [50,51]. PC2 for the dissolved phase, accounting for 7.2% of variability, is dominated by IDP plus Chr to a certain degree. Chr, according to Khuman et al. (2018), acts as a marker for coal combustion [38]. Additionally, a relatively (expressed as [IDP]/[IDP + BPe] ratio) high concentration of IDP would indicate pyrogenicity, coal burning in particular [36]. The implications of PC2 in particulate and dissolved phases over [Ant]/[Ant + Phe] and [IDP]/[IDP] + [BPe] ratios are detailed in Supplementary Materials.

### 3.4. Environmental Pathways

The mean values of Σ_14_PAH_part_ by area (west, central, east) are presented in Table 1. Σ_14_PAH_part_ is highest in the west area, decreasing towards the central and east areas (Figure 2). The west area also presents the highest mean turbidities and lowest mean salinities (Table 1, Figure 2, Figures S2 and S3). An inverse correlation was observed between turbidity and salinity. The r^2^ values for the five seasonal sampling expeditions were 0.976, 0.626, 0.945, 0.393, and 0.864, respectively. The lowest r^2^ values found in February can be attributed to snowfalls brought by winter monsoon. Less erosion and lower temperatures, limiting biological activity, would lower suspended solids concentrations, while melting snow would decrease salinity in a smoother gradient. To evaluate the correlation, turbidity versus Σ_14_PAH_part_ means by area were plotted, giving correlation coefficients (r^2^) of 0.999, 0.960, 0.947, 0.985, and 0.989 for each seasonal sampling expedition, respectively. Similarly, the anti-correlation of salinity versus Σ_14_PAH_part_ was evaluated by the r^2^ coefficients for their plots. The r^2^ coefficients were 0.979, 0.427, 0.999, 0.516, and 0.927, corresponding to each sampling expedition. May, November, and September presented stronger anti-correlations between salinity and Σ_14_PAH_part_ than August and February due to the overall lower salinity in all the areas of the bay due to summer and winter monsoon rainfalls.

The mean values of Σ_14_PAH_diss_ by area (west, central, east) are presented in Table 1. Mean Σ_14_PAH_diss_ variability among sampling areas did not always follow the west > central > east tendency shown by Σ_14_PAH_part_. The r^2^ coefficient sets for the turbidity–Σ_14_PAH_diss_ and salinity–Σ_14_PAH_diss_ plots were (0.118, 0.393, 0.924, 0.0986, 0.0062) and (0.0373, 0.0004, 0.998, 0.877, 0.086), corresponding to each sampling expedition. With longer retention times, compared with suspended solids, once inside the bay basin, the Σ_14_PAH_diss_ correlation with turbidity and salinity is less apparent [52,53].

August 2019 presented seven points with abnormally high dissolved PAH levels (>10 ng L^−1^) in the west and central areas. To solve the incongruity, seawater samples around the affected area were re-taken in the summer seasons that followed, together with water samples from the three river mouths (salinity 0–15 PSU) during both non-rain and rain events (rainfall: 52.5 mm day^−1^). Finally, the closest fishing port was also sampled.

Because the fishing port had relatively high Σ_14_PAHs (24.80 ng L^−1^) and Σ_14_PAH_diss_ over 5 times Σ_14_PAH_part_, the dissolved phase had a petrogenic character; therefore, the fishing port was discarded as the main pollution source. Σ_14_PAHs_diss_ from river water samples under normal conditions were all of a similar order of magnitude (12–21 ng L^−1^) and higher than most samples in the bay (Table S2). Thus, they can be considered as constant input concentrations during the summer season, but they were not necessarily responsible for the values recorded in August 2019. The Σ_14_PAHs in river water samples during rain events were an order of magnitude higher than average Σ_14_PAH_diss_ in West Nanao Bay. Together with the overall higher fluxes, rain events brought considerably great amounts of PAHs into the bay system. Daily rainfall data are presented in Figure S4, where it can be seen how nearly 200 mm of rainfall was recorded during one week before sampling in August 2019. With Σ_14_PAH_diss_ > 40 ng L^−1^ in all river water samples during rain events and the prolonged number of days of rainfall prior to sampling, the main pollution source in August 2019 is thus explained as being the result of runoff, followed by the respective water mixing–dilution. As demonstrated by Nakada et al., Kumaki and Ninomiya river plumes converge in the area between sampling points 11 and 3 during rain events (rainfall: 24 mm day^−1^) [54]. Despite no conclusive studies being found regarding PAH concentration variations within rain-induced runoff plumes, considerably higher PAH concentrations should be expected at headwaters after the initial “wash-out” of dried–deposited PAHs at the watershed. The Σ_14_PAHs_diss_ found at points 11 and 3 were as high as “river water during rain event” samples. Furthermore, during the rain events, riverine plumes transported greater amounts of eroded particles. The greater particle densities and sizes accelerated scavenging, explaining the low particulate phase percentage of Σ_14_PAHs in August (only 9.3%) and the poorer anti-correlation between salinity and Σ_14_PAH_part_.

### 3.5. Ecological Risk Assessment

Results of the assessment of ecological risk in surface seawater of West Nanao Bay are presented in Table 4. Some sampled points in the east area, such as B1 and 8, had low RQ_∑14PAHs(NCs)_ values, even equal to 0 in some seasons, representing very low to negligible risk. In contrast, many sampled points on the west side present RQ_∑14PAHs(NCs)_ values greater than 20 more than once. Sampling point C3, located at the Otsu River mouth, had RQ_∑14PAHs(NCs)_ values of 20.68, 18.79, 53.80, 17.86, and 84.53 in the five sampling surveys, becoming consistently the most polluted point in the bay. Although sampling point 12 at the Ninommiya river mouth also presented high RQ_∑14PAHs(NCs)_ values, sampling point 2 at the Kumaki river mouth was one of the less contaminated points in the west area of the bay. Thus, not all river mouths are necessarily high-risk areas. Differences in RQ_∑14PAHs(NCs)_ values reflect the differences in land use of the three watersheds. RQ_∑14PAHs(MPCs)_ was less than 1 for all sampled points in all sampled seasons.

RQ_NCs_ of individual PAHs varied from 0.026 (Chr, sampling point 3, May 2019) to 28.37 (BaA, sampling point C3, September 2020). BaA had RQ_NCs_ > 1, indicating a moderate risk to marine organisms, in 59 out of the 74 samples, being greater than 1 in all west and central areas’ points in all seasons and in all east areas’ points in November 2019. BaP and BPe also presented RQ_NCs_ > 1 in 49 and 48 samples, respectively. Sampling point 11 in August 2019 and C3 in September 2020 had the highest RQ_NCs_ (82.49 and 84.53, respectively). In the first one, 3- and 4-ring PAHs (Fle, Phe, Ant, Flu, Pyr, BaA) presented the major risks, of which BaA had the highest single PAH RQ_NCs_ (17.79). Next, 4–6-ring PAHs (Pyr, BaA, BbF, BPe) presented the major risks, of which BaA had the highest single PAH RQ_NCs_ (28.37). RQ_MPCs_, on the other hand, were less than 1 for all PAHs in all samples.

Finally, it is known that many polar PAH metabolites—such as 4-hydroxybenz[*a*]anthracene; 3-, 4-, and 10-hydroxybenz[*a*]anthracenes; and 2-hydroxychrysene—have TEF values up to 10 times greater than original PAHs, in which case, ecological risks would be sub-estimated [19,23,55]. Although these and many other polar PAHs were not considered, the method proposed by Cao et al. provides a simple way to assess risk based solely on the concentrations and toxic equivalent factors of the 16 US EPA-designated PAHs, providing valuable information on the corresponding monitored sites.

## 4. Conclusions

From May 2019 to September 2020, particulate and dissolved PAH average concentrations were repeatedly analyzed in a four-seasons-long monitoring survey performed on surface waters of West Nanao Bay, Japan. Mean total PAH concentrations were lower than 20.0 ng L^−1^ for most samples. Based on [Flu]/[Flu + Pyr] and [BaA]/[BaA + Chr] isomeric ratios and a varimax rotated PCA, it was established that biomass combustion was the principal source in the particulate phase, and liquid fossil fuel combustion was the principal source in the dissolved phase. From salinity and turbidity distribution, riverine runoffs were determined to be a major and continuing transportation pathway of particulate PAHs towards the bay. River water samples suggest that dissolved PAHs had the same input routes as particulate PAHs but within rain-dominated pulses. The risk quotients (RQ_∑14PAHs(NCs)_: 0–84.53) indicated that PAHs represented a very low to low environmental risk. BaA, BaP, and BPe were the three PAHs that presented single-component RQ_NCs_ > 1 in the greater number of samples. The most polluted sample and the one with the highest ecological risk was consistently sampling point C3, located in the west area of the bay, where direct riverine runoffs are present.

This work will directly contribute to studies of the chronic effects of PAHs on environments with low contamination levels (e.g., wildlife protected coastal areas) within Japan and overseas. As reliable field data, paradigms such as negligible concentrations could continue to encroach bodies of water, justifying future research in the sublethal toxic effects of PAHs. Finally, the understanding of PAHs’ environmental pathways in remote areas will contribute to improve remediation initiatives at the local level.

## Figures and Tables

**Table 1:** Salinity, turbidity, and particulate (Σ_14_PAH_part_) and dissolved (Σ_14_PAH_diss_) PAH averages ^a^ at each subarea, for each sampling season.

Sampling Season		2019			2020	
		May	August	November	February	September
West area (*n* = 7)	Salinity (PSU)	32.94 ± 0.12	30.70 ± 0.35	31.64 ± 0.64	31.33 ± 0.52	30.35 ± 0.21
	Turbidity (FTU)	1.03 ± 0.26	1.27 ± 0.24	1.50 ± 0.69	1.12 ± 0.28	2.45 ± 0.83
	Σ_14_PAH_part_ (ng L^−1^)	7.08 ± 3.94	2.27 ± 1.76	10.30 ± 5.09	5.15 ± 1.69	14.83 ± 7.68
	Σ_14_PAH_diss_ (ng L^−1^)	4.47 ± 0.50	9.16 ± 3.49	4.80 ± 0.99	4.29 ± 0.46	6.89 ± 1.64
Central area (*n* = 4)	Salinity (PSU)	33.04 ± 0.19	31.67 ± 0.26	32.54 ± 0.88	31.51 ± 0.96	31.16 ± 0.37
	Turbidity (FTU)	0.57 ± 0.15	0.94 ± 0.31	0.90 ± 0.19	0.77 ± 0.23	1.57 ± 0.77
	Σ_14_PAH_part_ (ng L^−1^)	1.14 ± 1.12	0.93 ± 0.51	4.79 ± 2.86	1.77 ± 1.18	3.99 ± 2.20
	Σ_14_PAH_diss_ (ng L^−1^)	5.30 ± 1.85	26.80 ± 18.63	2.77 ± 0.54	4.22 ± 1.25	5.72 ± 0.97
East area (*n* = 4)	Salinity (PSU)	33.02 ± 0.20	32.31 ± 0.14	32.8 ± 0.54	31.78 ± 0.99	31.74 ± 0.30
	Turbidity (FTU)	0.60 ± 0.09	1.03 ± 0.54	0.89 ± 0.16	0.86 ± 0.13	1.51 ± 0.82
	Σ_14_PAH_part_ (ng L^−1^)	1.57 ± 0.98	1.57 ± 2.32	2.15 ± 0.94	2.22 ± 1.61	1.75 ± 1.34
	Σ_14_PAH_diss_ (ng L^−1^)	5.67 ± 1.75	3.87 ± 0.99	1.41 ± 0.37	2.85 ± 1.89	7.61 ± 2.77

^a^ Averages by zone with their respective standard deviations.

**Table 2:** Diagnostic isomer ratio averages (± standard deviation) at each sample season, for particulate and dissolved PAHs.

Sampling Season		2019			2020	
		May	August	November	February	September
[Flu]/([Flu] + [Pyr]) ^a^	Σ_14_PAH_part_	0.63 ± 0.20	0.46 ± 0.06	0.40 ± 0.05	0.44 ± 0.03	0.42 ± 0.02
	Σ_14_PAH_diss_	0.75 ± 0.13	0.54 ± 0.11	0.51 ± 0.08	0.67 ± 0.04	0.42 ± 0.18
[BaA]/([BaA] + [Chr]) ^b^	Σ_14_PAH_part_	0.43 ± 0.05	0.37 ± 0.10	0.48 ± 0.06	0.42 ± 0.02	0.39 ± 0.31
	Σ_14_PAH_diss_	0.55 ± 0.07	0.62 ± 0.12	0.60 ± 0.10	0.41 ± 0.07	0.04 ± 0.04

^a^ fluoranthene (Flu) to pyrene isomeric ratio. ^b^ benzo[a]anthracene (BaA) to chrysene (Chr) isomeric ratio.

**Table 3:** Varimax normalized matrix of 14 PAHs in particulate and dissolved phase samples.

PAHs	Particulate Phase Principal Components		Dissolved Phase Principal Components	
	PC1	PC2	PC1	PC2
Ace	0.186	0.616	0.974	—
Fle	0.189	0.505	0.974	—
Phe	0.540	−0.563	0.951	0.100
Ant	0.847	0.415	0.975	—
Flu	0.940	0.273	0.973	—
Pyr	0.934	0.324	0.937	0.140
BaA	0.961	0.136	0.980	0.112
Chr	0.978	—	0.685	0.393
BbF	0.922	0.316	0.931	0.238
BkF	0.910	0.250	0.952	0.177
BaP	0.751	0.424	0.959	0.114
DBA	0.591	0.628	0.979	0.120
BPe	0.948	0.207	0.935	0.156
IDP	0.933	0.151	—	0.971
Estimated source	Pyrogenic	Petrogenic	Pyrogenic	Petrogenic
Variance (%)	71.0	9.2	84.5	7.2

**Table 4:** Risk quotients for negligible concentrations (RQ_NCs_) of total PAHs (Σ_14_PAHs). RQ_NCs_ sorted by sampling point and sampling season.

Sampling Point	2019			2020	
	May	August	November	February	September
C3	20.68	18.79	53.80	17.86	84.53
2	4.25	6.71	21.85	6.83	36.76
9	12.43	4.14	19.66	10.57	30.24
10	25.16	2.38	31.62	18.46	49.48
12	34.74	—	12.83	11.57	37.04
C4	20.04	7.49	21.33	10.68	31.83
A1	6.36	5.65	20.95	11.08	24.01
11	1.11	82.49	2.67	0.00	19.34
3	1.64	41.27	17.17	0.00	8.22
6	5.38	6.95	16.24	8.94	13.33
B2	0.00	5.62	5.80	1.23	1.41
B1	0.00	0.00	2.65	0.00	1.75
4	6.16	0.00	9.23	7.26	1.24
8	1.47	0.00	4.00	0.00	2.94
5	6.29	12.22	8.87	8.29	9.30

## Data Availability

Not applicable.

## Supplementary Materials

The following are available online at https://www.mdpi.com/article/10.3390/ijerph181910310/s1, Figure S1: Illustration of tandem filtration system adaptation for simultaneously separate particulate PAHs and perform solid phase extraction of dissolved PAHs. Figure S2: Silinity seasonal changes in surface water of West Nanao Bay, Japan; August 2019–September 2020. * different scale, Figure S3: Turbidity (FTU) seasonal changes in surface water of West Nanao Bay, Japan; August 2019–September 2020. * different scale. Figure S4: Daily rainfall (mm) before sampling surveys at observation station Nishiyachi (Kumaki river upstream). Pluvial data obtained from Ishikawa Prefectural Civil Engineering Department River Division. Table S1: Concentrations of the targeted 14 PAHs in the particulate phase (Σ14PAHpart) and dissolved phase (Σ14PAHdiss) at the 15 seasonally sampled points in West Nanao Bay during 2019–2020, Table S2: Concentrations of the targeted 14 PAHs in the particulate phase (Σ14PAHpart) and dissolved phase (Σ14PAHdiss) at end members in West Nanao Bay, Table S3: PAHs ecological risk assessment guideline, Table S4: Toxic equivalent factor, negligible concentrations, and maximum permitted concentrations for USEPA 16 priority PAHs.
