# Supplementary material for: Geochemical Control of PAHs by Inflowing River Water to West Nanao Bay, Japan, and Its Influences on Ecological Risk: Small-Scale Changes Observed under Near-Background Conditions at an Enclosed Bay
Source: Int J Environ Res Public Health. 2021 Sep 30;18(19):10310. doi: 10.3390/ijerph181910310 (PMC8508535; doi:10.3390/ijerph181910310)
Supplement: Supplementary file 1 [file ijerph-18-10310-s001.zip › ijerph-1362929-supplementary.pdf]

Supportive information

Geochemical Control of PAHs by Inflowing River Water to West Nanao Bay, Japan; and Its Influences on Ecological Risk: Small-Scale Changes Observed under Near-Background Conditions at an Enclosed Bay

Rodrigo Mundo [1], Tetsuya Matsunaka [2,*], Hisanori Iwai [2], Shinya Ochiai [2] and Seiya Nagao [2]

[1] Division of Material Chemistry, Graduate School of Natural Science and Technology, Kanazawa University, Kanazawa 920-1192, Japan; rodrigomundo12@gmail.com
[2] Low Level Radioactivity Laboratory, Institute of Nature and Environmental Technology, Kanazawa University, Nomi 923-1224, Japan; h-iwai@se.kanazawa-u.ac.jp (H.I.); sochiai@se.kanazawa-u.ac.jp (S.O.); seiya-nagao@se.kanazawa-u.ac.jp (S.N.)
* Correspondence: matsunaka@se.kanazawa-u.ac.jp; Tel.: +81-76-151-4440

Abbreviations list:

- US EPA: United States Environmental Protection Agency
- PAHs: polycyclic aromatic hydrocarbons
- HPLC: High performance liquid chromatography
- FLD: fluorescence detector
- Nap: naphtalene
- Ace: acenaphthene
- Fle: fluorene
- Ant: anthracene
- Phe: phenanthrene
- Flu: fluoranthene
- Pyr: pyrene
- BaA: benzo[a]anthracene
- Chr: chrysene
- BbF: benzo[b]fluoranthene
- BkF: benzo[k]fluoranthene
- BaP: benzo[a]pyrene
- BPe: benzo[ghi]perylene
- IDP: indeno[1,2,3-cd]pyrene
- DBA: dibenzo[a,h]anthracene
- Ex/Em: wavelength pares of excitation and emission
- PCA: Principal component analysis
- Σ_{14}PAHs: Sum of 14 targeted PAHs` total concentration (dissolved + particulate PAHs)
- $\Sigma_{14}PAH_{part}$: Sum of 14 targeted PAHs` particulate phase concentration
- $\Sigma_{14}PAH_{diss}$: Sum of 14 targeted PAHs` dissolved phase concentration
- LOD: Limit of detection
- RQ: Risk quotients
- QV: quality value
- NC: negligible concentration
- MPC: maximum permissible concentration
- TEF: toxic equivalent factors

Supplementary Methods

PAHs analysis. The HPLC system was configured as in Hayakawa *et al*. (2018) [23]. The system (Shimadzu co., Ltd. Japan) was equipped with two pumps (LC−20AB), auto injector (SIL−20A), and oven (CTO−20A) with columns: an Inertsil ODS−P analytical column (250 mm length, 4.6 mm internal diameter, 5 μm C18 100 Å; GL science Inc., Japan) and a guard column (10 mm length, 4.0 mm internal diameter, 5 μm C18 100 Å; GL science Inc., Japan) both kept at 30°C. Quantification and characterization of PAHs were carried out using a fluorescence detector (FLD) (SPD−20AV). The wavelength pairs of excitation and emission were operated at suitable values for each PAHs, described in previous studies [21−22]. The mobile phase was a mixture of acetonitrile and water operated under a gradient elution, starting at 55% and increasing to 99% acetonitrile over 65 min at a flow rate of 1 mL min^{-1}. Standards included the 16 PAHs in the EPA 610 mixture: naphthalene (Nap), acenaphthylene (Acy), acenaphthene (Ace), fluorene (Fle), phenanthrene (Phe), anthracene (Ant), fluoranthene (Flu), pyrene (Pyr), benz[*a*]anthracene (BaA), chrysene (Chr), benzo[*b*]fluoranthene (BbF), benzo[*k*]fluoranthene (BkF), benzo[*a*]pyrene (BaP), dibenzo[*ah*]anthracene (DBA), benzo[*ghi*]perylene (BgPe), and indeno[1,2,3−*cd*] pyrene (IDP). Due to low recoveries Nap could not be quantitatively measured and was thus excluded from the final analysis. Acy was not measurable with the current fluorescence detector. The concentrations of the remaining 14 PAH in particulate and dissolved phases ($\Sigma_{14}PAH_{part}$ and $\Sigma_{14}PAH_{diss}$) were added to provide the total 14 PAHs concentrations (Σ_{14}PAHs) in the marine samples.

Table S1. Concentrations of the targeted 14 PAHs in the particulate phase ($\Sigma_{14}PAH_{part}$) and dissolved phase ($\Sigma_{14}PAH_{diss}$) at the 15 seasonally sampled points in West Nanao Bay during 2019–2020

		PAHs Concentrations (ng L^{-1})														
		C3	2	9	10	12	C4	A1	11	3	6	B2	B1	4	8	5
May 23rd, 2019	$\Sigma_{14}PAH_{part}$	7.92	2.54	4.89	10.26	13.35	7.73	2.87	0.40	0.69	2.82	0.65	0.83	2.38	0.61	2.45
	$\Sigma_{14}PAH_{diss}$	4.49	3.64	4.72	4.17	5.17	4.25	4.83	4.19	6.92	6.83	3.27	3.42	7.66	6.13	5.47
August 27th, 2019	$\Sigma_{14}PAH_{part}$	5.68	0.76	1.54	1.25	-	2.47	1.91	1.11	0.25	1.49	0.88	0.59	0.45	0.19	5.04
	$\Sigma_{14}PAH_{diss}$	10.44	14.22	7.18	6.62	-	4.94	11.55	48.83	35.64	10.44	12.29	2.57	3.62	4.75	4.53
November 13th, 2019	$\Sigma_{14}PAH_{part}$	21.01	9.16	7.85	11.76	5.31	8.28	8.72	1.79	7.30	7.18	2.90	1.19	2.73	1.50	3.17
	$\Sigma_{14}PAH_{diss}$	5.39	3.26	4.77	5.12	3.85	6.26	4.93	2.93	2.96	3.22	1.97	1.13	1.53	1.09	1.90
February 7th, 2020	$\Sigma_{14}PAH_{part}$	7.36	2.80	4.20	7.42	4.94	4.40	4.89	1.11	1.11	3.53	1.34	0.39	3.45	1.33	3.70
	$\Sigma_{14}PAH_{diss}$	4.31	4.40	3.99	3.97	5.12	4.55	3.72	3.58	5.34	5.20	2.77	2.12	3.77	0.60	4.91
September 30th, 2020	$\Sigma_{14}PAH_{part}$	30.66	11.99	10.42	18.79	12.38	11.06	8.50	6.42	3.33	4.92	1.29	1.36	1.07	0.82	3.73
	$\Sigma_{14}PAH_{diss}$	7.43	9.11	6.02	4.81	5.84	8.91	6.10	6.50	4.34	6.26	5.78	6.58	5.11	11.55	7.20

Table S2. Concentrations of the targeted 14 PAHs in the particulate phase ($\Sigma_{14}PAH_{part}$) and dissolved phase ($\Sigma_{14}PAH_{diss}$) at end members in West Nanao Bay

Condition	Site	Phase	PAHs Concentrations (ng L^{-1})
Non rain	Kumaki	$\Sigma_{14}PAH_{part}$	1.12
		$\Sigma_{14}PAH_{diss}$	17.71
	Ninomiya	$\Sigma_{14}PAH_{part}$	9.52
		$\Sigma_{14}PAH_{diss}$	12.27
	Otsu	$\Sigma_{14}PAH_{part}$	0.46
		$\Sigma_{14}PAH_{diss}$	20.56
Rain	Kumaki	$\Sigma_{14}PAH_{part}$	85.02
		$\Sigma_{14}PAH_{diss}$	51.76
	Ninomiya	$\Sigma_{14}PAH_{part}$	11.85
		$\Sigma_{14}PAH_{diss}$	40.79
	Otsu	$\Sigma_{14}PAH_{part}$	6.04
		$\Sigma_{14}PAH_{diss}$	44.74
Non rain	Fishing port	$\Sigma_{14}PAH_{part}$	4.41
		$\Sigma_{14}PAH_{diss}$	20.39

Table S3. PAHs ecological risk assessment guideline [26].

		Water (ng L^{-1})	
PAHs	TEFs$^{\alpha}$	NCs$^{\beta}$	MPCs$^{\gamma}$
Naphthalene	0.001	12	1200
Acenaphthene	0.001	3	300
Fluorene	0.001	3	300
Phenanthrene	0.001	3	300
Anthracene	0.01	0.7	70
Fluoranthene	0.001	3	300
Pyrene	0.001	0.7	70
Benzo[a]anthracene	0.1	0.1	10
Chrysene	0.01	3.4	340
Benzo[b]fluoranthene	0.1	0.4	40
Benzo[k]fluoranthene	0.1	0.4	40
Benzo[a]pyrene	1	0.5	50
Dibenzo [a, h] anthracene	1	0.5	50
Benzo[ghi]perylene	0.01	0.3	30
Indeno[1,2,2-cd] pyrene	0.01	0.3	30
$\sum_{14}$PAHs	—	31$^{\delta}$	3100$^{\varepsilon}$

$^{\alpha}$ Toxicity equivalent factors

$^{\beta}$ The quality values of the negligible concentrations for individual PAHs

$^{\gamma}$ The quality value for the maximum permissible concentrations for individual PAHs

$^{\delta}$ The total quality value of negligible concentrations for 14 PAHs targeted in this study

$^{\varepsilon}$ The total quality value of the maximum permissible concentrations for 14 PAHs targeted in this study

Table S4. Toxic equivalent factor, negligible concentrations, and maximum permitted concentrations for USEPA 16 priority PAHs [24].

For Individual PAHs			For ∑PAHs		
	$RQ_{(NCs)}^{\alpha}$	$RQ_{(MPCs)}^{\beta}$		$RQ_{\sum PAHs\ (NCs)}^{\gamma}$	$RQ_{\sum PAHs\ (MPCs)}^{\delta}$
Very low risk	0	—	Very low risk	～0	—
			Low-risk	≧1;<800	0
Moderate-risk	≧1	<1	Moderate-risk 1	≧800	0
			Moderate-risk2	<800	≧1
High-risk	—	≦1	High-risk	≧800	≧1

$^{\alpha}$ Toxic equivalent factors

$^{\beta}$ The negligible concentrations for individual PAHs

$^{\gamma}$ The maximum permissible concentrations for individual PAHs

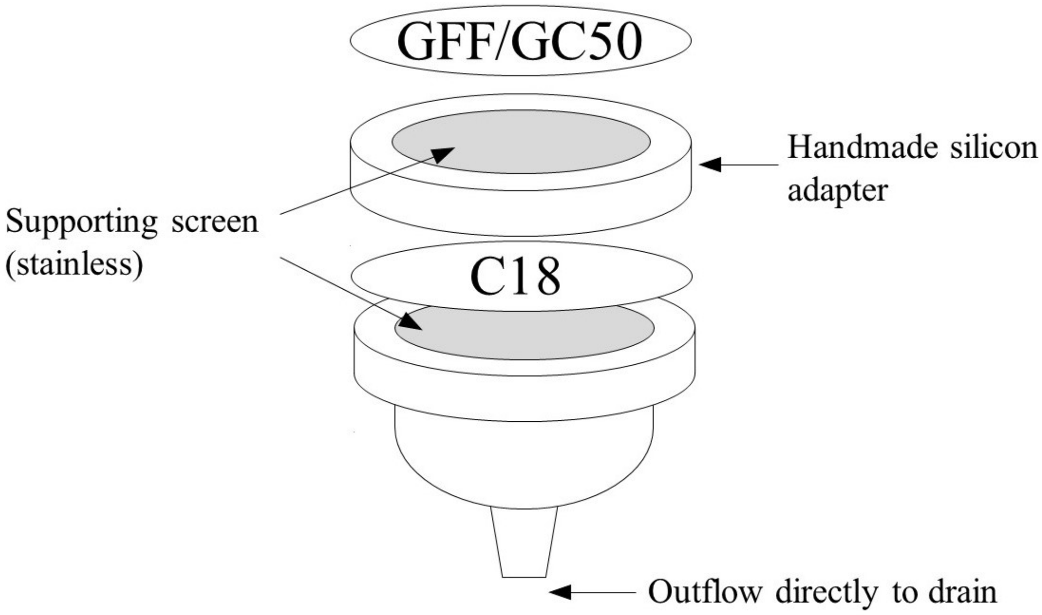

Figure S1. Illustration of tandem filtration system adaptation for simultaneously separate particulate PAHs and perform solid phase extraction of dissolved PAHs.

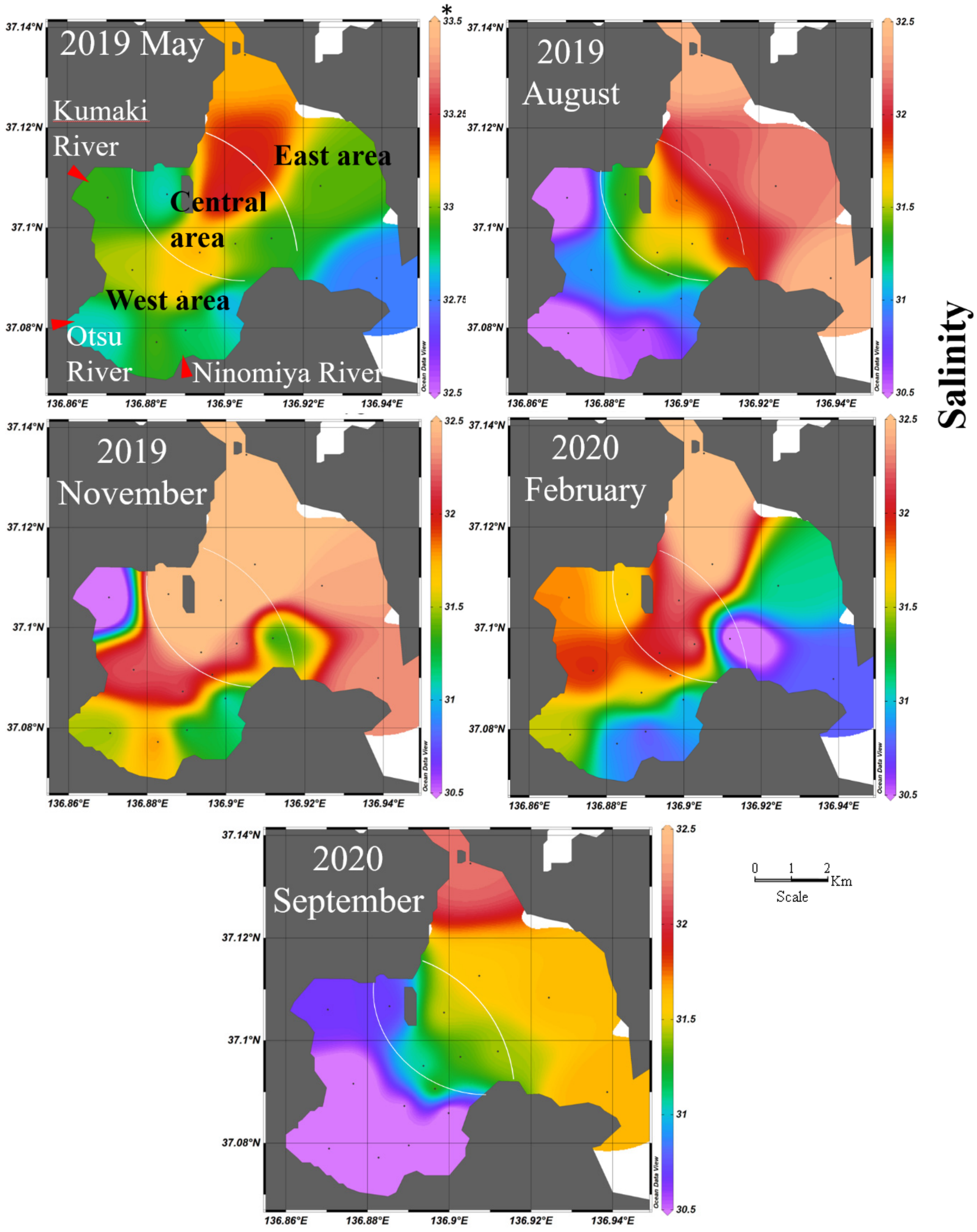

Figure S2. Silinity seasonal changes in surface water of West Nanao Bay, Japan; August 2019-September 2020. * different scale

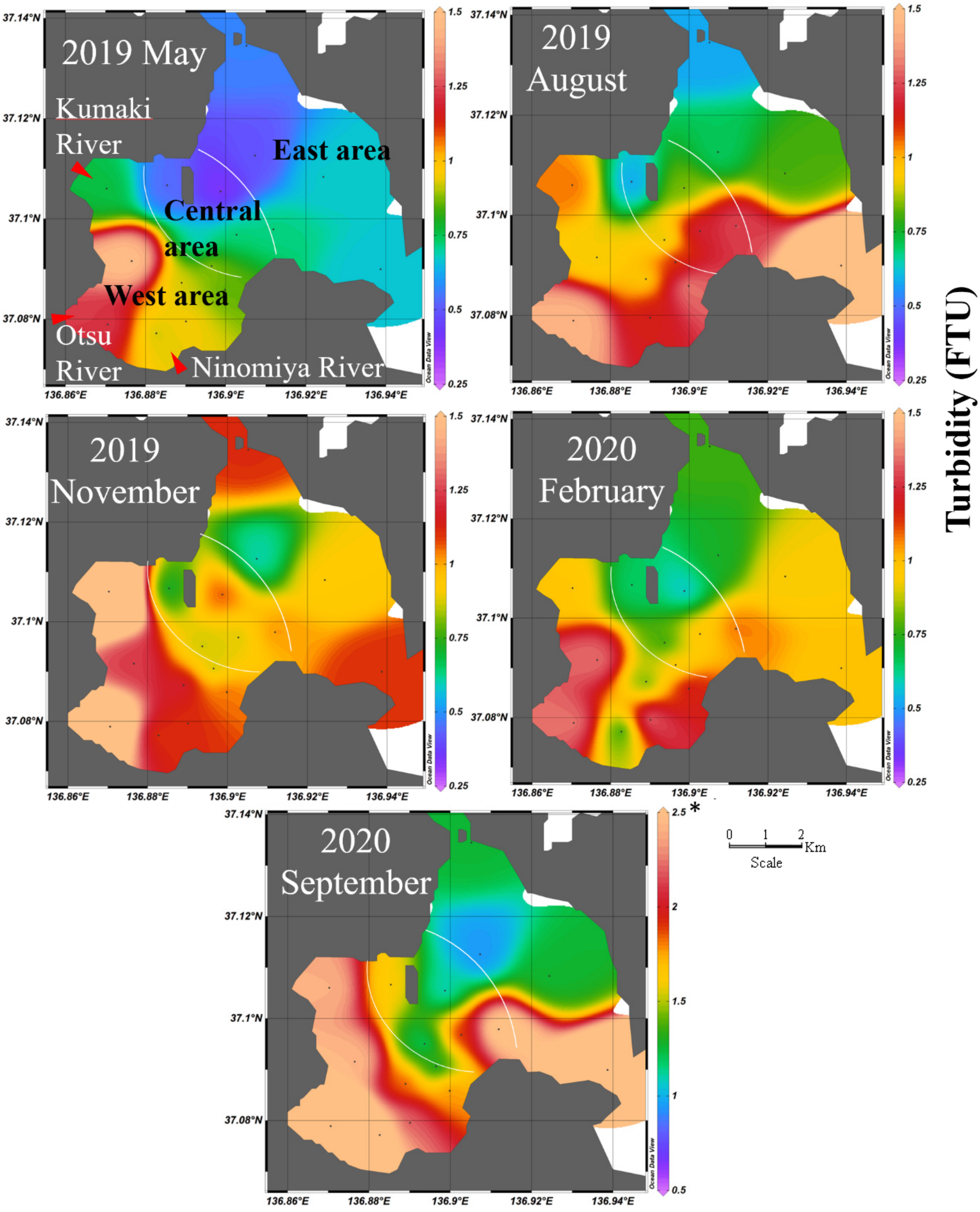

Figure S3. Turbidity (FTU) seasonal changes in surface water of West Nanao Bay, Japan; August 2019-September 2020. * different scale

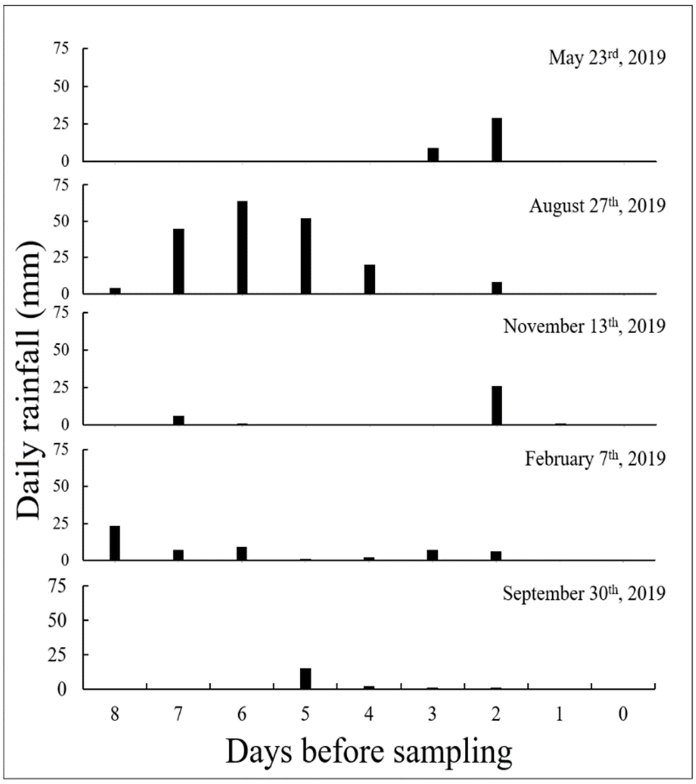

Figure S4. Daily rainfall (mm) before sampling surveys at observation station Nishiyachi (Kumaki river upstream). Pluvial data obtained from Ishikawa Prefectural Civil Engineering Department River Division.
